# Illumination matters part I: comparative analysis of light sources and illumination in flexible ureteroscopy-fundamental findings from a PEARLS analysis

**DOI:** 10.1007/s00345-024-05037-7

**Published:** 2024-05-26

**Authors:** Jia-Lun Kwok, Vincent De Coninck, Mariela Corrales, Alba Sierra, Frédéric Panthier, Eugenio Ventimiglia, Vineet Gauhar, Florian Alexander Schmid, Manuela Hunziker, Cédric Poyet, Daniel Eberli, Olivier Traxer, Etienne Xavier Keller

**Affiliations:** 1 Department of Urology, University Hospital Zurich, University of Zurich, Zurich, Switzerland; 2 Department of Urology, Tan Tock Seng Hospital, Singapore, Singapore; 3 Progressive Endourological Association for Research and Leading Solutions (PEARLS), Paris, France; 4 Young Academic Urologists (YAU), Endourology & Urolithiasis Working Group, Arnhem, The Netherlands; 5 Department of Urology, AZ Klina, Brasschaat, Belgium; 6 GRC N°20, Groupe de Recherche Clinique sur la Lithiase Urinaire, Sorbonne Université, Hôpital Tenon, F-75020 Paris, France; 7 Urology Department, Hospital Clinic de Barcelona, Villarroel 170, 08036 Barcelona, Spain; 8 Division of Experimental Oncology/Unit of Urology, Urological Research Institute, IRCCS Ospedale San Raffaele, Milan, Italy; 9 Department of Urology, Ng Teng Fong General Hospital, Singapore, Singapore

**Keywords:** Flexible ureteroscopy, Light properties, Illumination, Saline, Air

## Abstract

**Purpose:**

Illumination characteristics of flexible ureteroscopes have been evaluated in air, but not in saline, the native operative medium for endourology. The aim was to evaluate light properties of contemporary ureteroscopes in air versus saline, light distribution analysis, and color temperature.

**Methods:**

We evaluated the Storz Flex-Xc and Flex-X2s, Olympus V3 and P7, Pusen 7.5F and 9.2F, and OTU WiScope using a 3D printed black target board in-vitro model submerged in saline. A spectrometer was used for lux and color temperature measurements at different opening locations.

**Results:**

Illuminance was higher in saline compared to air (5679 vs. 5205 lx with Flex-Xc, p = 0.02). Illuminance in saline differed between ureteroscopes (ANOVA p < 0.001), with highest for the Flex-Xc at 100% brightness setting (5679 lx), followed by Pusen 9.2F (5280 lx), Flex-X2s (4613 lx), P7 (4371 lx), V3 (2374 lx), WiScope (582 lx) and finally Pusen 7.5F (255 lx). The same ranking was found at 50% brightness setting, with the highest ureteroscope illuminance value 34 times that of the scope with lowest illuminance.

Most scopes had maximum illuminance off center, with skewness. Three scopes had two light sources, with one light source for all other scopes. Inter-scope comparisons revealed significant differences of color temperature (ANOVA p < 0.001).

**Conclusion:**

The study demonstrates the presence of inhomogeneous light spread as well as large differences in illumination properties of ureteroscopes, possibly impacting on the performance of individual scopes in vivo. Additionally, the study suggests that future studies on illumination characteristics of flexible ureteroscopes should ideally be done in saline, and no longer in air.

**Supplementary Information:**

The online version contains supplementary material available at 10.1007/s00345-024-05037-7.

## Introduction

Ureteroscopy is currently the most frequent intervention for renal stones in many countries worldwide, and is an established procedure commonly done in urology practice [1, 2], with a plethora of applications [3]

Imaging characteristics of ureteroscopes impacting on clarity, visibility, contrast, color accuracy and resolution are important parameters that have been extensively evaluated [4–10] due to their importance in treatment efficacy and safety. Image clarity allows accurate identification of pathology, precise stone lasering, basketing, biopsy and ablation of urothelial carcinoma.

Vision and clarity of images in ureteroscopy are dependent on many factors, including illumination and distribution of light from the tip of ureteroscopes. With newer and single-use ureteroscopes, homogenous and comparable illumination characteristics would be expected from scopes available for use in daily clinical routine. On the contrary, there have been previous reports on undesirable artefacts from poor light source design causing shadowing and dark corners in the endoscopic field of view [8], which ideally should not arise in scopes that were already released for use in humans.

Only few studies so far have evaluated light source of ureteroscopes, and all were performed at fixed distances with scopes held in air instead of saline [6, 7, 11–15]. One study looked at light intensity distribution characteristics in prototype ureteroscope tip models, but exact illuminance was not measured [16]. Illumination properties depend on the light source from the scope and the medium it is projected across. To the best of our knowledge, no prior study on ureteroscope optics was done in saline (NaCl 0.9%), which is in fact the native ureteroscopy medium used in clinical routine. The refraction index differs between air and saline [17], and illumination properties arguably may vary accordingly.

The aim of this present study was to evaluate illumination properties of contemporary ureteroscopes, including comparisons in air vs. saline. Light sources from ureteroscopes were also evaluated in an analysis of the tip design.

## Material and methods

We evaluated a series of currently available flexible ureteroscopes at our institution, including the Flex-Xc and Flex-X2s (Karl Storz SE & Co. KG, Tuttlingen, Germany), URF-P7 and URF-V3 (Olympus, Centre Valley, PA, USA), Uscope 7.5F PU3033A and Uscope 9.2F PU3022A (Zhuhai Pusen Medical Technology Co. Ltd. Guangdong, China) as well as the OTU WiScope (OTU Medical Inc, CA, USA). To get as close as possible to real working conditions, the single-use scopes (Pusen 7.5F, Pusen 9.2F and OTU WiScope) were brand new models from sealed sterilized packages. Reusable scopes (Storz and Olympus scopes) had all been rinsed, disinfected, and decontaminated after use in humans, with no record of the number of prior interventions.

For the Storz Flex-X2s, the Power LED 175 light source (unit used < 100 h) was used with a corresponding 230 cm and 3.5 mm fiberoptic cable, as well as with the IMAGE1 S HX-P HDTV 1-Chip pendular camera (Karl Storz SE & Co. KG, Tuttlingen, Germany). For the Olympus URF-P7 and URF-V3, the VISERA elite CLV-S190 light source (Xenon short-arc lamp used < 100 h) was used together with a corresponding WA03310A 300 cm and 4.3 mm fiberoptic light cable, as well as the CH S190 08 LB camera head (Olympus, Centre Valley, PA, USA). Both fiberoptic cables were brand new.

A color spectrometer incorporating the Vishay VEML 6040 color sensor (RGBW200, ELV Elektronik AG, Leer, Germany) was used for lux and color temperature measurements, as well as for red, green and blue (RGB) light measurements. The Vishay VEML6040 color sensor senses RGB as well as white light with peak sensitivities for RGB at 645 nm, 575 nm, and 460 nm, respectively, according to the manufacturer information [18]. The range of spectral bandwidth for RGB is ± 45 nm, ± 45 nm, and ± 35 nm, respectively*.* The high sensitivity and wide dynamic range of the sensor allows for light intensity measurements between < 0.01 lx up to 16’496 lx. RGB values are provided as normalized values relative to the reading settings of the sensor (% of maximum). The sensor was waterproofed with a transparent polyvinylidene chloride film to allow experiments to be performed in saline (NaCl 0.9%).

**Fig. 1:**
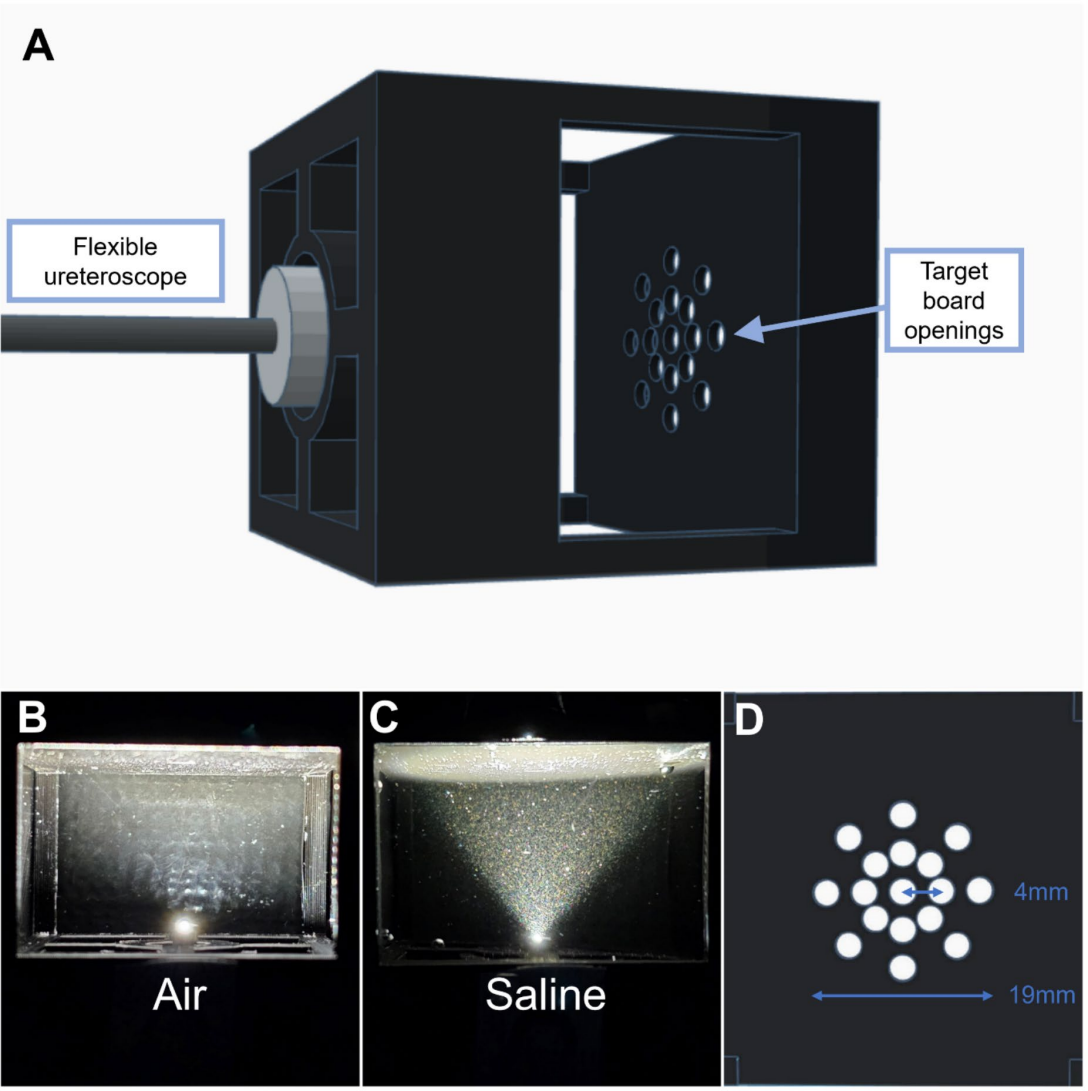
Experimental setup for illumination characteristics of ureteroscopes. **A** Experimental setup with a 3D printed black model; **B **and **C** Top view of the Flex-Xc illumination in air and saline; **D** Target board layout with center, inner and outer ring openings

A 3D printed black model was used to hold the ureteroscopes at a fixed distance of 20 mm from the center of a target board in a dark room (Fig. 1A). The target board included one center opening, as well as eight inner and eight outer ring openings distributed in circles at 4 mm and 8 mm from the center opening respectively, resulting in a 19 mm diameter target circle. The ureteroscope was maintained in a non-deflected, straight position, with the center of the endoscopic view aligned to the center opening of the target board. The size of the target field and distance from the light sensor was chosen with reference to dimensions of models constructed on data from endocasts [19] used in studies testing scopes in the setting of laser lithotripsy [20], to reflect in vivo settings. The tray holding the set-up was colored black to minimize noise from scattered light and all measurements were performed in a black chamber.

For each measurement, the color spectrum analyzer sensor was placed at one of the 17 openings of the target board and measurements were repeated 5 times. For each repeated measurement, the ureteroscope was withdrawn and reinserted into the 3D printed black model. Illuminance was measured at all 17 target board openings, while color temperature and RGB measurements were only performed for the center opening. We arbitrarily chose the Storz Flex-Xc to compare light properties in air vs. saline at 50% and 100% brightness settings. Afterwards, the entire experiment set-up was performed in saline including all scopes, thus replicating usual conditions found in clinical routine during ureteroscopy.

Whenever the scope setup allowed for light source brightness adjustment, measurements were repeated at light brightness settings of 50% and 100%. For the Storz Flex-Xc this was found available to be adjusted only via the menu buttons on the scope handle. For the Storz Flex-X2s, in addition to brightness settings adjustable on the light source unit, buttons on the camera head also allowed for separate brightness adjustment. Preliminary analysis of the Storz Flex-Xc results led the authors to decide for the Storz Flex-X2s to keep the brightness setting in the camera head at 50% and take only measurements for the 50% and 100% brightness settings adjusted on the light stack. For all other scopes, the brightness setting was adjusted on the light stack. Since light brightness can be set either to automatic or manual mode on the Olympus light stack as well as with the Storz camera head, all measurements were performed using the manual mode to ensure consistent measurements throughout all experiments.

Finally, ureteroscope tip designs were evaluated. Digital still photographs of the tip design of ureteroscopes were taken with an Olympus Pen E-P7 camera and a Panasonic Leica DG Macro-Elmarit 45 mm 2.8 lens. The angulations between the ureteroscope lens, light source(s) and working channel were measured using the computer software *ImageJ* [21].

## Statistical analysis

Illuminance was expressed as mean values of 5 repeated measurement in lux. For skewness, a clock face view of the endoscopic scope image was used for easy reference and direct application to daily clinical practice. For calculation of skewness, the mean value resulting from the inner and outer opening at each clock face direction was used. From there, the result was expressed as percentage of the clock face direction with the highest illuminance, allowing to draw a radar chart for each scope.

An analysis on all scopes at 50% and 100% brightness settings was performed using one-way ANOVA with Tukey post hoc comparisons. Student’s t-test analyses were performed to evaluate the impact of air vs. saline, brightness settings and distance of openings from center on mean illuminance, color temperature and RGB values*.* For all tests, a two-sided p value < 0.05 was considered statistically significant. All statistical tests were performed with GraphPad Prism 9.5.1 (GraphPad Software, La Jolla CA, USA). Illuminance maps were created using mean illuminance values with the web program *Heatmapper* [22]. Radar charts of the light illuminance skewness were made with Microsoft Excel for Microsoft 365 MSO Version 2302 Build 16.0.16130.20332 (Microsoft Corporation, Redmond WA, USA).

## Results

### Air vs. saline

Both overall illuminance and center opening illuminance were found to be significantly higher in saline compared to air (5679 vs. 5205 lx, p = 0.02 and 8600 vs. 6558 lx, p < 0.001, respectively) (Table 1). Additionally, there was a significant illuminance drop between the inner and outer ring measurements in air (−660 lx, p < 0.005), whereas no significant drop was found in saline (p = 0.23). The top view of the light illumination path was noted to be comparatively more dispersed in air, while rather triangular with distinct borders in saline (Fig. 1B and C).

**Table 1:** Illumination properties of flexible ureteroscopes

Air versus saline: storz flex-xc							
	Illuminance at 100% brightness setting (lux)		Mean increase (lux) (95% CI)	Percentage Increase		p-value^b^	
	Air	Saline					
Mean overall (95% CI)^a^	5205 (5062 to 5349)	5679 (5298 to 6059)	473 (70 to 877)	9%		p = 0.02	
Maximum at center of target board?	Yes	Yes	NA	NA		NA	
Mean at center opening (95% CI)	6558 (6247 to 6870)	8600 (8480 to 8719)	2041 (1764 to 2318)	31%		p < 0.001	
Mean at inner ring openings (95% CI)	5451 (5277 to 5625)	5721 (5271 to 6171)	270 (−205 to 745)	5%		p = 0.26	
Mean at outer ring openings (95% CI)	4791 (4655 to 4926)	5271 (4677 to 5865)	481 (−119 to 1080)	10%		p = 0.11	
Mean change between inner and outer ring openings (95% CI)	−660 (−877 to −443)	−450 (−1183 to 284)	NA	−12% (air)	−8% (saline)	p < 0.01 (air)	p = 0.23 (saline)

Saline: mean overall illuminance of flexible ureteroscopes								
Scope		50% brightness setting		100% brightness setting		Mean increase (lux) (95% CI)	Percentage Increase	p-value^b^
		Mean^a^ (lux) (95% CI)	Relative factor to scope with lowest illuminance	Mean^a^ (lux) (95% CI)	Relative factor to scope with lowest illuminance			
Reusable	Storz Flex-Xc	5504 (5120 to 5887)	34 times	5679 (5298 to 6059)	22 times	175 (−361 to 711)	3%	p = 0.52
	Storz Flex-X2s	3240 (3058 to 3422)	20 times	4613 (4370 to 4857)	18 times	1374 (1072 to 1675)	42%	p < 0.001
	Olympus V3	664 (544 to 785)	4 times	2374 (1983 to 2765)	9 times	1710 (1303 to 2116)	258%	p < 0.001
	Olympus P7	2216 (1888 to 2543)	14 times	4371 (3813 to 4930)	17 times	2156 (1513 to 2799)	97%	p < 0.001
Single-use	Pusen 7.5F	160 (146 to 174)	–	255 (233 to 276)	–	95 (69 to 120)	59%	p < 0.001
	Pusen 9.2F	3406 (3079 to 3733)	21 times	5280 (4777 to 5783)	21 times	1874 (1278 to 2470)	55%	p < 0.001
	OTU WiScope	288 (265 to 311)	2 times	582 (534 to 630)	2 times	294 (241 to 347)	102%	p < 0.001

^a^Considering all measurements over all target board openings

^b^Student’s T-test

### Illumination characteristics in saline

Overall illuminance significantly differed between different flexible ureteroscopes at both 50% and 100% brightness settings (ANOVA p < 0.001 for both settings) (Table 1).

The Flex-Xc had the highest overall illuminance at 100% brightness settings (5679 lx), followed by Pusen 9.2F (5280 lx at 100% brightness settings), Flex-X2s (4613 lx), P7 (4371 lx), V3 (2374 lx), WiScope (582 lx) and finally the Pusen 7.5F with the lowest overall illuminance readings (255 lx). The same ranking was found for 50% brightness settings (Table 1).

For each scope, there was a significant increase in mean overall illuminance when comparing the 50% and 100% brightness settings, with the highest relative overall illuminance increase for the V3 (+ 258%), and the lowest relative overall illuminance increase with the Flex-X2s (+ 42%). One exception was the Flex-Xc where no significant difference between the 50% and 100% setting was found.

**Fig. 2:**
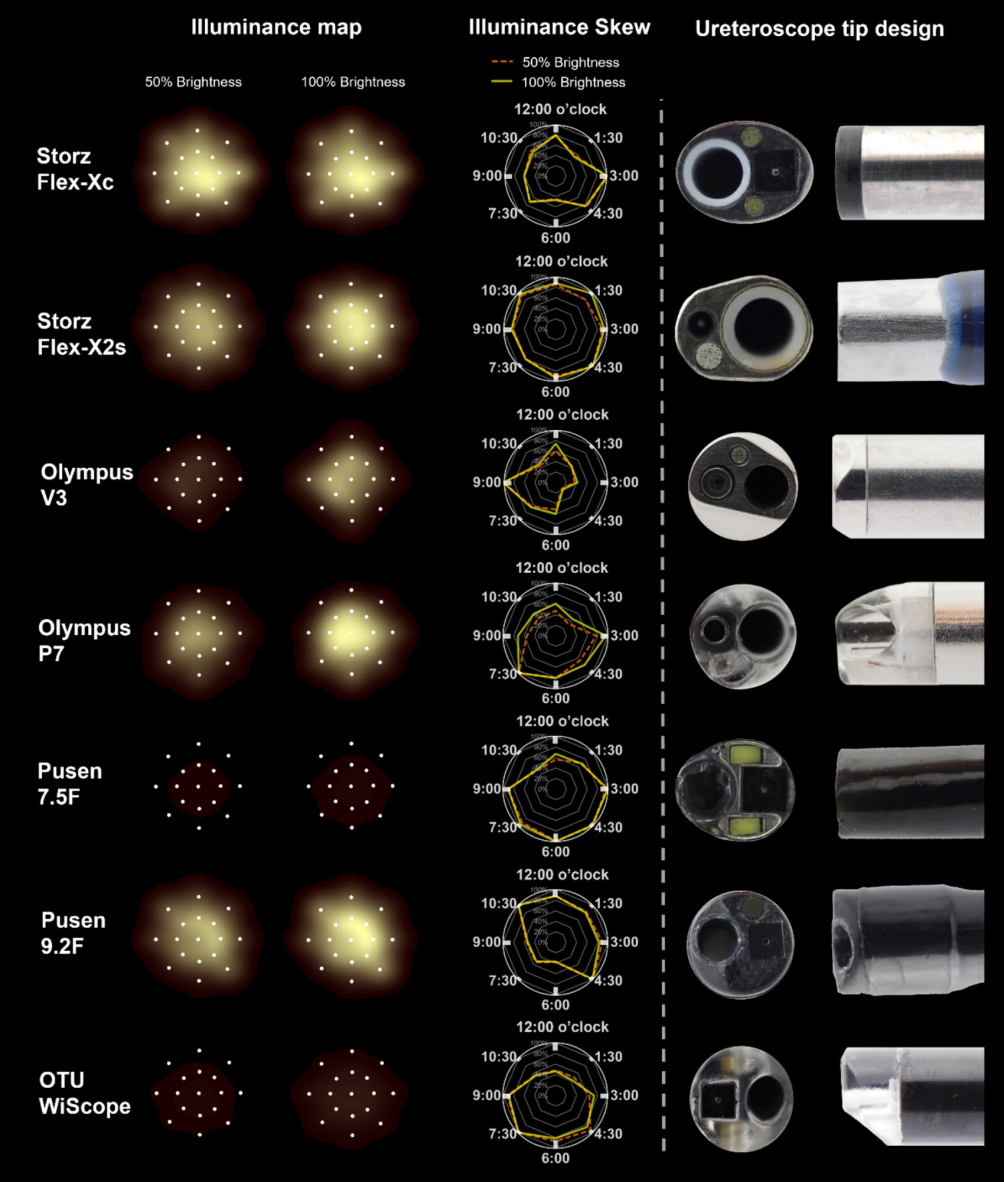
Illuminance maps, skewness in saline and tip design of flexible ureteroscopes. Illuminance and skewness maps are represented from an endoscopic point of view, with center of the endoscopic view aligned to the black model center opening. Each white point on the illuminance map corresponds to an opening on the black model. Ureteroscope tip configurations are front facing (according to endoscopic view) and side facing photographs (scope rotated to illustrate beveled tip

Only two out of seven scopes had their point of maximum illuminance consistently at the center opening of the target board: the Flex-X2s and P7 (Table 2, Fig. 2). All other scopes had maximum illuminance off-center at 50% and/or 100% brightness setting.

**Table 2:** Illuminance spread over a target board in saline

Scope	50% brightness setting								100% brightness setting							
	Maximum illuminance		Direction of maximum skewness from center	Mean illuminance (lux)					Maximum Illuminance		Direction of maximum skewness from center	Mean illuminance (lux)				
	Target board opening location	Mean (lux) (95% CI)		Inner ring openings (95% CI)	Outer ring openings (95% CI)	Mean change of outer vs. inner ring (95% CI)	Percentage change of outer vs. inner ring	p-value^a^	Target board opening location	Mean (lux) (95% CI)		Inner ring openings (95% CI)	Outer ring openings (95% CI)	Mean change of outer vs. inner ring (95% CI)	Percentage change of outer vs. inner ring	p-value^a^
Reusable																
Storz Flex-Xc	3.00 o’clock outer ring	8622 (8363 to 8882)	3.00 o’clock	5569 (5128 to 6010)	5059 (4461 to 5657)	−509 (−1241 to 222)	−9%	p = 0.17	Centre	8600 (8480 to 8719)	3.00 o’clock	5721 (5271 to 6171)	5271 (4677 to 5865)	−450 (−1183 to 284)	−8%	p = 0.23
Storz Flex-X2s	Centre	4544 (4421 to 4667)	4.30 o’clock	3860 (3723 to 3997)	2457 (2352 to 2561)	−1403 (−1573 to -1233)	−36%	p < 0.001	Centre	6146 (6026 to 6266)	4.30 o’clock	5469 (5267 to 5671)	3566 (3437 to 3694)	−1903 (−2139 to −1668)	−35%	p < 0.001
Olympus V3	9.00 o’clock inner ring	1693 (1644 to 1741)	9.00 o’clock	1095 (965 to 1225)	186 (105 to 266)	−910 (−1060 to −759)	−83%	p < 0.001	9.00 o’clock inner ring	5245 (5120 to 5370)	9.00 o’clock	3757 (3350 to 4164)	849 (539 to 1158)	−2908 (−3411 to −2405)	−77%	p < 0.001
Olympus P7	Centre	5262 (4605 to 1741)	7.30 o’clock	2950 (2676 to 3223)	1101 (751 to 1451)	−1849 (−2286 to −1412)	−63%	p < 0.001	Centre	8017 (7911 to 8123)	7.30 o’clock	6064 (5606 to 6522)	2223 (1667 to 2778)	−3841 (−4550 to −3133)	−63%	p < 0.001
Single-use																
Pusen 7.5F	Centre	223 (207 to 240)	4.30 o’clock	199 (194 to 204)	113 (92 to 134)	−86 (−107 to −66)	−43%	p < 0.001	12.00 o’clock inner ring	345 (336 to 354)	6.00 o’clock	318 (312 to 324)	182 (149 to 215)	−136 (−169 to −103)	−43%	p < 0.001
Pusen 9.2F	3.00 o’clock inner ring	6282 (6230 to 6333)	4.30 o’clock	4249 (3849 to 4649)	2587 (2140 to 3034)	−1662 (−2252 to −1071)	−39%	p < 0.001	3.00 o’clock inner ring	9209 (9091 to 9328)	10.30 o’clock	6497 (5901 to 7092)	4058 (3329 to 4787)	−2439 (−3366 to −1512)	−38%	p < 0.001
OTU WiScope	4.30 o’clock inner ring	417 (410 to 425)	7.30 o’clock	341 (325 to 257)	232 (192 to 271)	−109 (−152 to −67)	−32%	p < 0.001	4.30 o’clock inner ring	845 (840 to 851)	7.30 o’clock	707 (675 to 738)	453 (372 to 533)	−254 (−339 to −169)	−36%	p < 0.001

^a^Student’s T-test comparing illuminance for inner vs outer ring

There was a significant drop in mean overall illuminance between inner ring (4 mm away from center) and outer ring (8 mm away from center) for all scopes, except for the Flex-Xc where no significant differences were found (Table 2, Fig. 2). The highest relative drop between inner and outer ring measurements was found for the V3 (−83% at 50% brightness setting, and −77% at 100% brightness setting, respectively). There was presence of different illuminance skewness patterns across scopes (Fig. 2).

### Color temperature

Color temperature measurement results are shown in Supplementary Fig. 1. Intra-scope comparisons between 50 and 100% brightness revealed significant differences for three out of seven scopes: + 838 Kelvin (95% CI 40 to 1637; p = 0.04) for the V3 from 50 to 100% light source brightness settings, −223 Kelvin (95% CI −408 to −39; p = 0.02) for the P7 and −510 Kelvin (95% CI −912 to −108; p = 0.02) for the WiScope.

Inter-scope comparisons revealed significant differences of mean color temperature measurements (ANOVA p < 0.001). The lowest color temperature readings were found for the V3 (3322 Kelvin at 50%), which was significantly lower compared to all other scopes in the Tukey’s post hoc analysis (all p < 0.001). The two scopes with the highest color temperature reading ranges were the Pusen 7.5F (7502 Kelvin at 50%) and the WiScope (8159 Kelvin at 50%), which were both significantly higher compared to all other scopes in the Tukey’s post hoc analysis (all p < 0.001).

### Red green blue (RGB) normalized values

The maximum channel for the normalized RGB mean measurements was consistently green for all scopes, except for red with the V3 (Supplementary Table 1). The minimum channel was blue for all scopes across 50% and 100% brightness settings. When comparing across scopes, the respective RGB values for all scopes were significantly different at both 50% and 100% brightness settings (both ANOVA p < 0.001).

When comparing individual Red, Green and Blue channel differences at 50% vs 100% brightness settings within each scope, all RGB measurements were significantly different except for the Red and Blue readings for the Flex-Xc (Supplementary Table 1).

### Ureteroscope tip design

Ureteroscope tip designs differ widely between scopes (Fig. 2). Of relevance to illumination, three scopes had two light sources (Flex-Xc, Pusen 9.2F, and the WiScope), while the rest had only one light source (Table 3). The light source position relative to the optical lens varied widely as well. Interestingly, two scopes (P7 and WiScope) had transparent tips, with the end of the light sources contained within the transparent part of the tip. All the rest of the scopes had the light source level with the end of the scope tip. All scopes were found to have fiberoptic light sources except two scopes with light emitting diode (LED) sources at the tip of the scope (Pusen 9.2F and WiScope).

**Table 3:** Characteristics of flexible ureteroscopes’ tips and light sources

Scope	Number of separate light sources	Light source position relative to optical lens from endoscopic view perspective	Working channel position relative to the optical lens from endoscopic view perspective	Transparent tip of scope	Light source tip at same level with end of scope	Shape of tip (from front view)	Size (Fr)^a^		Tip Beveled	Light at tip: fiberoptic vs. LED	Brightness setting adjustment location	Light source location
							Tip	Shaft				
Reusable												
Storz Flex-Xc	2	1 & 5 o’clock	3 o’clock	No	Yes	Oval	8.5	8.4	No	Fiberoptic	Scope handle menu	In scope
Storz Flex-X2s	1	6.30 o’clock	9 o’clock	No	Yes	Oval	7.5	7.5	Yes	Fiberoptic	Camera head / Light stack	Light stack
Olympus V3	1	10 o’clock	9 o’clock	No	Yes	Circular	8.5	8.4	Yes	Fiberoptic	Light stack	Light stack
Olympus P7	1	6.30 o’clock	9 o’clock	Yes	No	Circular	4.9	7.95	Yes	Fiberoptic	Light stack	Light stack
Single-use												
Pusen 7.5F	2	1 & 5 o’clock	3 o’clock	No	Yes	Oval	7.5	7.5	No	LED	Processing unit	In scope
Pusen 9.2F	1	1 o’clock	3 o’clock	No	Yes	Circular	9.2	9.2	Yes	Fiberoptic	Processing unit	In scope
OTU WiScope	2	11 & 7 o’clock	9 o’clock	Yes	No	Circular	7.4	8.6	Yes	LED	Processing unit	In scope

^a^Per manufacturer specifications

## Discussion

The difference in illumination properties of ureteroscopes in air and saline is likely due to the respective differing refractive indexes, in combination with absorption and scattering effects [17]. The findings of this study are important in suggesting that studies on ureteroscope illuminance should ideally not be done in air. The authors suggest that all such future studies should ideally be done in saline instead, to replicate the real performance conditions in endourological surgery.

We note that the Flex-Xc scope handle menu brightness settings of 50 and 100% had illuminance values which were not significantly different. We conclude that the actual illuminance of the light source did not change with different brightness settings, and this is a feature of the scope to increase the exposure of the image on the viewing screen.

Illuminance varies widely between ureteroscopes. The Flex-Xc had the highest illuminance across all scopes (both reusable and single-use). Out of the single-use ureteroscopes, the Pusen 9.2F had illuminance readings higher than other reusable ureteroscopes. We note the possibility of single-use scopes to have the capability of high illumination characteristics. On the other hand, the Pusen 7.5F had the lowest illuminance of all scopes, with a factor up to 34 times lower than the scope with the strongest illuminance, reaching merely 3% illuminance compared to the Flex-Xc. How this may impact in clinical routine needs to be evaluated in dedicated in vivo studies. Arguably, such differences in illumination characteristics may impact the overall clarity of the image of ureteroscopes and may partly explain the differences in image quality perception found in a prior study [23].

Urologists need to be aware of the skewness of light from ureteroscopes to prevent missing pathology in darker areas. This is especially valid in situations of poorer visibility including bleeding [23], larger hydronephrotic systems, or working at further distances from the target object. Clock face sectors with highest illuminance may correlate to the choice of scopes in right and left sided surgery, akin to working channel position [24]. The impact of skewness in clinical situations needs to be further evaluated in future studies.

With regards to color temperature readings, the V3 and P7 produced warmer light, while the Pusen 7.5F and WiScope produced cooler light. Clinical relevance of this finding is not clear and deserve dedicated investigation.

It is interesting that individual RGB values change between 50 and 100% brightness settings for all scopes except for the Flex-Xc (likely due to the very homogenous spread of light from its light source). All scopes had green as the maximum channel except for the Olympus V3.

It is important to note that with re-usable equipment, illuminance may deteriorate with time. After each procedure, the quality of vision may progressively worsen due to damage of the optical systems (illumination and imaging [25]), and additionally with fiberoptic ureteroscopes deterioration of the light cable and light source. The light stack should undergo maintenance regularly, and light cables renewed periodically. This is a fundamental difference between re-usable and single-use scopes, where single-use ureteroscopes are always optimal as brand new with each use.

The special transparent tip designs of the WiScope and P7 could be hypothesized to give a “omni-light” with a more homogenous spread of light. However, on the contrary our study showed significant drop in illuminance between inner and outer ring openings for the two scopes. The advantages or limitations of such transparent tip design needs to be further evaluated.

The study has several potential limitations. First, light measurements were taken in a ring-like fashion across the target board, with areas in between openings not measured. The target board area of 19 mm may not capture the entire fall of peripheral light. However, the study setup adequately samples a target field size and ureteroscope distance representative of in vivo ureteroscope use. Measurements wider to this target field are not as clinically relevant. Second, the reusable scopes used for this study were not brand new. However, the usage of the scopes reflects real life clinical scenarios as mentioned, and in fact in some instances had higher illuminance than brand new single-use scopes. Third, the present study is an in vitro attempt to assess ureteroscope properties that may impact in vivo surgery. The interpretation of the data must therefore be taken with care since environmental factors arguably may impact on clinical translation of the findings of this study. Black was used to decrease peripheral light scatter. However, this does not reflect the color of the renal collecting system. The experimental setup was performed as an open setup, which is not reflective of the closed renal collecting system cavity with effects of indirect and direct light. Measurements were also taken at a fixed 20 mm distance, and future studies could further investigate effects on different distances to the ureteroscope tip. Fourth, urine and blood may change illumination characteristics when present in addition to saline irrigation. This may change the refractive index as well as transmission of light across the saline-urine-blood interface and should be further investigated in future studies. Additionally, it is not clear how illuminance properties may impact the final video projection of the surgical site from ureteroscopes on operative monitors. Finally, while there is a wide range of scopes clinically available, we were only able to test a set number of reusable and single-use scopes available to the institution. Future studies could include other newer scopes that have since been made commercially available. Nonetheless, this does not change the main findings of our study: that illuminance varies very widely between different ureteroscopes.

Other aspects of ureteroscope illumination properties should be further evaluated in future studies, including the impact of illuminance and light source design on unwanted interferences (flashes or image artefacts) [26]. Additionally, it remains to be seen if the study findings can be useful in the design of future ureteroscopes with high resolution integrating Artificial Intelligence [27, 28].

## Conclusions

In line with previous reports on possible shortcomings of illuminance from flexible ureteroscopes, the present study demonstrates the presence of inhomogeneous light spread as well as large differences in illumination properties of ureteroscopes, possibly impacting on the performance of individual scopes in vivo. Additionally, illumination properties of ureteroscopes are different in air than in saline. The authors suggest that future studies on illumination characteristics of flexible ureteroscopes should ideally be done in saline, and no longer in air. Urologists should be aware of the non-centered maximum illuminance and skewness of some ureteroscopes. Observations from the present study deserve analysis in future studies, including situations in a closed cavity system [29], partial obstruction [30], varying effects of light brightness [31] and account for environmental factors such as urine and blood.

## Supplementary Information

Below is the link to the electronic supplementary material.

Supplementary file1 (TIF 16523 KB)

Supplementary file2 (DOCX 15 KB)

## Data Availability

On request to corresponding author for raw data on the experimental setup.

## References

[1] Geraghty RM, Jones P, Somani BK (2017) Worldwide trends of urinary stone disease treatment over the last two decades: a systematic review. J Endourol 31(6):547–556. 10.1089/end.2016.0895 28095709 10.1089/end.2016.0895

[2] Heers H, Stay D, Wiesmann T, Hofmann R (2022) Urolithiasis in Germany: trends from the national DRG database. Urol Int 106(6):589–595. 10.1159/000520372 34883491 10.1159/000520372 PMC9248299

[3] Giusti G, Proietti S, Peschechera R, Taverna G, Sortino G, Cindolo L et al (2015) Sky is no limit for ureteroscopy: extending the indications and special circumstances. World J Urol 33(2):257–273. 10.1007/s00345-014-1345-y 24962930 10.1007/s00345-014-1345-y

[4] Proietti S, Dragos L, Molina W, Doizi S, Giusti G, Traxer O (2016) Comparison of new single-use digital flexible ureteroscope versus nondisposable fiber optic and digital ureteroscope in a cadaveric model. J Endourol 30(6):655–659. 10.1089/end.2016.0051 27084572 10.1089/end.2016.0051 PMC4913498

[5] Lusch A, Abdelshehid C, Hidas G, Osann KE, Okhunov Z, McDougall E et al (2013) In vitro and in vivo comparison of optics and performance of a distal sensor ureteroscope versus a standard fiberoptic ureteroscope. J Endourol 27(7):896–902. 10.1089/end.2013.0003 23402369 10.1089/end.2013.0003 PMC3826566

[6] Hendriks N, Henderickx MMEL, Schout BMA, Baard J, van Etten-Jamaludin FS, Beerlage HP et al (2021) How to evaluate a flexible ureterorenoscope? systematic mapping of existing evaluation methods. BJU Int 128(4):408–423. 10.1111/bju.15544 34242475 10.1111/bju.15544 PMC8519042

[7] Bader MJ, Gratzke C, Walther S, Schlenker B, Tilki D, Hocaoglu Y et al (2010) The polyscope: a modular design, semidisposable flexible ureterorenoscope system. J Endourol 24(7):1061–1066. 10.1089/end.2010.0077 20575699 10.1089/end.2010.0077

[8] Dragos LB, Somani BK, Keller EX, De Coninck VMJ, Herrero MRM, Kamphuis GM et al (2019) Characteristics of current digital single-use flexible ureteroscopes versus their reusable counterparts: an in-vitro comparative analysis. Transl Androl Urol 8:S359–S370. 10.21037/tau.2019.09.17 31656742 10.21037/tau.2019.09.17 PMC6790413

[9] Doizi S, Kamphuis G, Giusti G, Andreassen KH, Knoll T, Osther PJ et al (2017) First clinical evaluation of a new single-use flexible ureteroscope (LithoVue™): a European prospective multicentric feasibility study. World J Urol 35(5):809–818. 10.1007/s00345-016-1936-x 27671898 10.1007/s00345-016-1936-x

[10] Schlager D, Obaid MA, Hein S, Wilhelm K, Schönthaler M, Gratzke C et al (2020) Current disposable ureteroscopes: performance and limitations in a standardized kidney model. J Endourol 34(10):1015–1020. 10.1089/end.2020.0185 32475165 10.1089/end.2020.0185

[11] Patil A, Agrawal S, Batra R, Singh A, Ganpule A, Sabnis R et al (2023) Single-use flexible ureteroscopes: comparative in vitro analysis of four scopes. Asian J Urol 10(1):64–69. 10.1016/j.ajur.2022.02.001 36721687 10.1016/j.ajur.2022.02.001 PMC9875117

[12] Deininger S, Haberstock L, Kruck S, Neumann E, da Costa IA, Todenhöfer T et al (2018) Single-use versus reusable ureterorenoscopes for retrograde intrarenal surgery (RIRS): systematic comparative analysis of physical and optical properties in three different devices. World J Urol 36(12):2059–2063. 10.1007/s00345-018-2365-9 29869701 10.1007/s00345-018-2365-9

[13] Paffen MLJE, Keizer JG, De Winter GV, Arends AJ, Hendrikx AJM (2008) A comparison of the physical properties of four new generation flexible ureteroscopes: (de)flection, flow properties, torsion stiffness, and optical characteristics. J Endourol 22(10):2227–2234. 10.1089/end.2008.0371 18831670 10.1089/end.2008.0371

[14] Afane JS, Olweny EO, Bercowsky E, Sundaram CP, Dunn MD, Shalhav AL et al (2000) Flexible ureteroscopes: a single center evaluation of the durability and function of the new endoscopes smaller than 9fr. J Urol 164(4):1164–1168. 10.1016/S0022-5347(05)67133-9 10992358

[15] Abdelshehid C, Ahlering MT, Chou D, Park HK, Basillote J, Lee D et al (2005) Comparison of flexible ureteroscopes: deflection, irrigant flow and optical characteristics. J Urol 173(6):2017–2021. 10.1097/01.ju.0000158139.65771.0a 15879808 10.1097/01.ju.0000158139.65771.0a

[16] Wilson CR, Kennedy JD, Irby PB, Fried NM (2018) Miniature ureteroscope distal tip designs for potential use in thulium fiber laser lithotripsy. J Biomed Opt. 10.1117/1.JBO.23.7.076003 29981222 10.1117/1.JBO.23.7.076003

[17] Zhuang S, Ji Y, Tu D, Zhang X (2022) Underwater RGB-D camera based on binocular stereo vision. Guangzi Xuebao/Acta Photonica Sinica 51(4):161–175. 10.3788/gzxb20225104.0404003

[18] Schaar R: 2023. https://www.vishay.com/docs/84331/designingveml6040.pdf. Accessed 31 March 2023

[19] Marroig B, Favorito LA, Fortes MA, Sampaio FJB (2015) Lower pole anatomy and mid-renal-zone classification applied to flexible ureteroscopy: experimental study using human three-dimensional endocasts. Surg Radiol Anat 37(10):1243–1249. 10.1007/s00276-015-1503-y 26044783 10.1007/s00276-015-1503-y

[20] Aldoukhi AH, Roberts WW, Hall TL, Teichman JMH, Ghani KR (2018) Understanding the popcorn effect during holmium laser lithotripsy for dusting. Urology 122:52–57. 10.1016/j.urology.2018.08.031 30195011 10.1016/j.urology.2018.08.031

[21] Rasband WS. 2018 U. S. National Institutes of Health, Bethesda, Maryland, USA1997–. p. ImageJ.

[22] Babicki S, Arndt D, Marcu A, Liang Y, Grant JR, Maciejewski A et al (2016) Heatmapper: web-enabled heat mapping for all. Nucleic Acids Res 44(1):W147–W153. 10.1093/NAR/GKW419 27190236 10.1093/nar/gkw419 PMC4987948

[23] Talso M, Proietti S, Emiliani E, Gallioli A, Dragos L, Orosa A et al (2018) Comparison of flexible ureterorenoscope quality of vision: an in vitro study. J Endourol 32(6):523–528. 10.1089/end.2017.0838 29562765 10.1089/end.2017.0838

[24] Villa L, Ventimiglia E, Proietti S, Giusti G, Briganti A, Salonia A et al (2020) Does working channel position influence the effectiveness of flexible ureteroscopy? results from an in vitro study. BJU Int 125(3):449–456. 10.1111/bju.14923 31610080 10.1111/bju.14923

[25] Traxer O, Dubosq F, Jamali K, Gattegno B, Thibault P (2006) New-generation flexible ureterorenoscopes are more durable than previous ones. Urology 68(2):276–279. 10.1016/j.urology.2006.02.043 16904434 10.1016/j.urology.2006.02.043

[26] Miller CS, Whiles BB, Ito WE, Machen E, Thompson JA, Duchene DA et al (2023) Image distortion during flexible ureteroscopy: a laboratory model comparing super pulsed thulium fiber laser vs high-power HO:YAG laser. J Endourol 37(1):99–104. 10.1089/end.2022.0195 36106599 10.1089/end.2022.0195 PMC10623464

[27] Estrade V, Daudon M, Richard E, Bernhard JC, Bladou F, Robert G et al (2022) Towards automatic recognition of pure and mixed stones using intra-operative endoscopic digital images. BJU Int 129(2):234–242. 10.1111/bju.15515 34133814 10.1111/bju.15515 PMC9292712

[28] Zeeshan Hameed BM, Aiswarya Dhavileswarapu VLS, Raza SZ, Karimi H, Khanuja HS, Shetty DK et al (2021) Artificial intelligence and its impact on urological diseases and management: a comprehensive review of the literature. J Clin Med. 10.3390/jcm10091864 10.3390/jcm10091864 PMC8123407 33925767

[29] Kwok J-L, Panthier F, De Coninck V, Ventimiglia E, Barghouthy Y, Danilovic A et al (2024) Illumination matters part II: advanced comparative analysis of flexible ureteroscopes in a kidney model by pearls. World J Urol 42(1):298. 10.1007/s00345-024-04987-2 38709327 10.1007/s00345-024-04987-2 PMC11074033

[30] Kwok J-L, Ventimiglia E, De Coninck V, Sierra A, Panthier F, Corrales M et al (2024) Illumination matters part III: impact of light obstruction on illuminance from flexible ureteroscopes - a comparative PEARLS analysis. World J Urol 42(1):188. 10.1007/s00345-024-04910-9 38520528 10.1007/s00345-024-04910-9 PMC10960769

[31] Kwok J-L, De Coninck V, Panthier F, Kamkoum H, Pauchard F, Shrestha A et al (2024) Illumination matters part IV: blackout and whiteout in flexible ureteroscopy - first report on a phenomenon observed by PEARLS. World J Urol. 42(1):294. 10.1007/s00345-024-04988-1 38704777 10.1007/s00345-024-04988-1 PMC11070394

