# Supplementary figures and images for: Illumination matters part I: comparative analysis of light sources and illumination in flexible ureteroscopy-fundamental findings from a PEARLS analysis
Source: World J Urol. 2024 May 26;42(1):355. doi: 10.1007/s00345-024-05037-7 (PMC11128383; doi:10.1007/s00345-024-05037-7)

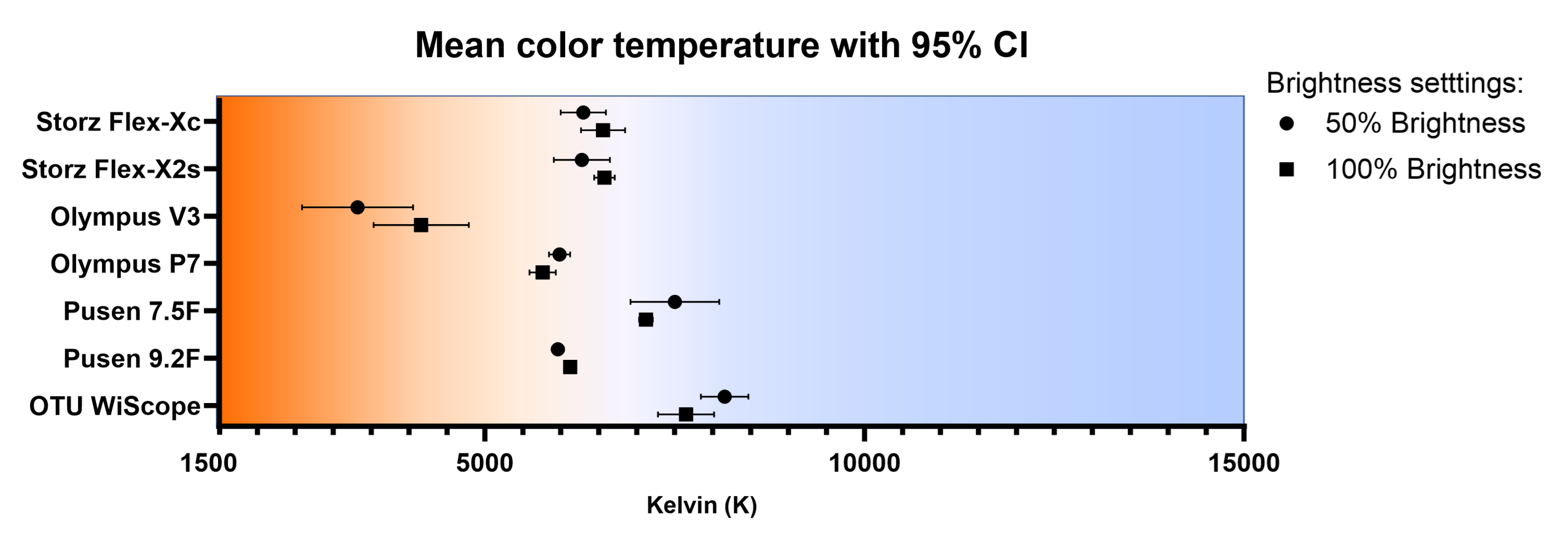

Supplement: Supplementary file 1 — Supplementary file1 (TIF 16523 KB) [file 345_2024_5037_MOESM1_ESM.tif]
