# Supplementary material for: Illumination matters part I: comparative analysis of light sources and illumination in flexible ureteroscopy-fundamental findings from a PEARLS analysis
Source: World J Urol. 2024 May 26;42(1):355. doi: 10.1007/s00345-024-05037-7 (PMC11128383; doi:10.1007/s00345-024-05037-7)
Supplement: Supplementary file 2 — Supplementary file2 (DOCX 15 KB) [file 345_2024_5037_MOESM2_ESM.docx]

| **Supplementary Table 1. Mean RGB values of flexible ureteroscopes at center target opening in saline** | | | | | | | | |
| --- | --- | --- | --- | --- | --- | --- | --- | --- |
|  | 50% brightness setting  (95% CI) | | | | 100% brightness setting  (95% CI) | | | |
| Scope | Red | Green | Blue | Max | Red | Green | Blue | Max |
| Storz Flex-Xc | 37 (36 to 38) | 51 (49 to 52) | 25 (24 to 27) | Green | 37 (35 to 38) | 52 (52 to 53) | 26 (26 to 27) | Green |
| Storz Flex-X2s | 37 (36 to 39) | 54 (53 to 56) | 24 (23 to 24) | Green | 52 (50 to 53) | 74 (73 to 74) | 27 (36 to 37) | Green |
| Olympus V3 | 17 (13 to 21) | 13 (12 to 14) | 6 (6 to 7) | Red | 44 (42 to 47) | 42 (38 to 46) | 20 (15 to 25) | Red |
| Olympus P7 | 49 (42 to 55) | 63 (55 to 71) | 32 (28 to 36) | Green | 41 (40 to 42) | 49 (48 to 49) | 27 (25 to 29) | Green |
| Pusen 7.5F | 13 (11 to 14) | 21 (20 to 23) | 10 (9 to 10) | Green | 20 (19 to 21) | 31 (30 to 33) | 15 (25 to 29) | Green |
| Pusen 9.2F | 15 (14 to 15) | 20 (19 to 20) | 10 (9 to 10) | Green | 24 (24 to 25) | 33 (32 to 34) | 16 (16 to 17) | Green |
| OTU WiScope | 11 (10 to 13) | 15 (13 to 17) | 10 (8 to 12) | Green | 20 (16 to 23) | 28 (26 to 29) | 18 (17 to 18) | Green |
| p-value | p<0.001 | p<0.001 | p<0.001 | - | p<0.001 | p<0.001 | p<0.001 | - |
| *One-way ANOVA of mean RGB values for each scope | | | | | | | | |
